# Impact of Rituximab on Remission Rates in Granulomatosis With Polyangiitis: A Systematic Review

**DOI:** 10.7759/cureus.66838

**Published:** 2024-08-14

**Authors:** Papuna Papuashvili, Giorgi Vepkhishvili, Tinatin Makaridze, Giorgi Popiashvili

**Affiliations:** 1 Internal Medicine, Tbilisi State Medical University, Tbilisi, GEO; 2 Oncology, Todua Clinic, Tbilisi, GEO; 3 Internal Medicine, American Hospital Tbilisi, Tbilisi, GEO; 4 Internal Medicine, Gudushauri Clinic, Tbilisi, GEO

**Keywords:** randomized controlled trials, anca-associated vasculitis, remission, rituximab, wegener's granulomatosis, gpa, granulomatosis with polyangiitis

## Abstract

This systematic review evaluates the efficacy of rituximab in inducing and maintaining remission in patients with granulomatosis with polyangiitis (GPA). We conducted a comprehensive search across multiple databases, identifying 81 studies, of which 11 met our inclusion criteria after rigorous screening and assessment for relevance and quality. Our analysis shows that rituximab, compared to traditional treatments such as cyclophosphamide and azathioprine, significantly improves remission rates and reduces relapse frequency in GPA patients. Notably, rituximab's benefits extend across various patient demographics, including pediatric groups, and are evident in different dosing regimens, highlighting its versatility and potential as a first-line therapy. The review also underscores the importance of personalized medicine approaches in managing GPA, as rituximab's effectiveness was particularly pronounced in patients with relapsing disease forms. Future research should focus on long-term outcomes, optimal dosing strategies, and the economic implications of widespread rituximab use in clinical practice. Our findings advocate for the integration of rituximab into standard treatment protocols for GPA, offering new hope for patients afflicted with this challenging autoimmune disorder.

## Introduction and background

Granulomatosis with polyangiitis (GPA), formerly known as Wegener's granulomatosis, is a form of anti-neutrophil cytoplasmic antibody (ANCA)-associated vasculitis that predominantly affects the respiratory tract and the kidneys, leading to potentially life-threatening complications if left untreated [1]. Traditional management strategies have heavily relied on the use of cyclophosphamide and glucocorticoids, which, while effective in inducing remission, are associated with significant toxicities and a high relapse rate [2]. In recent years, rituximab, a chimeric monoclonal antibody against CD20, has emerged as a promising alternative, potentially revolutionizing the therapeutic landscape of GPA by offering a mechanism-based approach to deplete B lymphocytes, cells instrumental in the pathogenesis of the disease [3].

The advent of rituximab has prompted a re-evaluation of treatment paradigms, particularly given its success in other autoimmune conditions and its favorable safety profile compared to traditional cytotoxic therapies [4]. The shift from conventional immunosuppression to targeted B-cell depletion represents not just a therapeutic alternative but a paradigm shift in understanding and managing autoimmune vasculitis [5]. This review seeks to dissect the breadth of evidence amassed from clinical trials that have explored rituximab's role in inducing and maintaining remission in patients with GPA, emphasizing comparing its efficacy and safety against established regimens.

The primary objective of this systematic review is to critically analyze and synthesize existing research concerning the impact of rituximab on remission rates in patients with granulomatosis with polyangiitis. This review aims to delineate the efficacy of rituximab not only in achieving disease remission but also in maintaining it, thereby assessing its potential as a superior mainstay treatment for GPA. Furthermore, it intends to explore patient outcomes, relapse rates, and adverse events associated with rituximab therapy, comparing these aspects with those of traditional therapies such as cyclophosphamide. Through this comprehensive analysis, this review seeks to provide a robust academic resource that could guide clinical decisions and influence future research directions in the management of GPA.

## Review

Materials and methods

Search Strategy

Our search strategy was rigorously developed in line with the Preferred Reporting Items for Systematic Reviews and Meta-Analyses (PRISMA) guidelines [6] to meticulously explore the role of rituximab in the management of granulomatosis with polyangiitis (GPA). Comprehensive searches were conducted across a spectrum of key biomedical databases including PubMed, Medline, Embase, the Cochrane Library, and Scopus. Our search timeframe extended from the inception of each database up to June 2024, ensuring the inclusion of the most current research.

We utilized a combination of keywords and Medical Subject Headings (MeSH) terms specifically chosen to align with our research objectives. These terms included "granulomatosis with polyangiitis," "Wegener's granulomatosis," "rituximab," "remission," "ANCA-associated vasculitis," and "randomized controlled trials." Boolean operators ("AND" and "OR") were employed to structure complex search strings that could effectively intersect the diverse aspects of our research focus. Representative search queries included "granulomatosis with polyangiitis AND rituximab AND remission," "Wegener's granulomatosis AND therapeutic use of rituximab," and "rituximab OR B lymphocyte depletion AND efficacy in ANCA-associated vasculitis."

To expand the scope of our search and ensure a comprehensive literature capture, we also reviewed the reference lists of all retrieved articles for additional relevant studies. Moreover, our search was extended to include clinical trial registries and pertinent conference proceedings to uncover unpublished or ongoing studies that may provide valuable insights into emerging therapies and their efficacies.

To maintain high academic and clinical rigor, our search was limited to studies published in English and peer-reviewed journals. The inclusion criteria were specifically designed to capture clinical trials, cohort studies, and randomized controlled trials (RCTs) that evaluate the efficacy of rituximab in inducing and maintaining remission in patients diagnosed with GPA. An expert in medical literature retrieval, specializing in autoimmune diseases and therapeutic interventions, reviewed our search strategy to ensure its robustness and comprehensiveness.

Eligibility Criteria

The eligibility criteria for this systematic review have been established to ensure the scientific rigor and clinical relevance of the included studies. We focus exclusively on peer-reviewed research articles that evaluate the efficacy of rituximab in treating granulomatosis with polyangiitis (GPA). Eligible studies include clinical trials, randomized controlled trials (RCTs), cohort studies, and meta-analyses that provide empirical evidence on rituximab's effectiveness in inducing and maintaining remission in GPA patients. These studies must detail clinical outcomes following rituximab administration, either as a primary treatment or in comparison to other agents such as cyclophosphamide or azathioprine, and must involve patients diagnosed with ANCA-associated vasculitis, specifically GPA. Additionally, only studies published in English from the databases' inception until June 2024 are included.

Conversely, the exclusion criteria omit articles not meeting the review's stringent requirements. Studies that do not specifically address rituximab's role in GPA or focus on other vasculitis forms without clear relevance to rituximab's effect on GPA are excluded. Non-peer-reviewed articles, case reports, editorials, and commentary pieces are also excluded to maintain a focus on high-quality empirical research. Furthermore, studies lacking rigorous methodological details, those without specific outcome measures relevant to remission induction or maintenance, and articles not published in English are also excluded to ensure clarity and consistency in data interpretation and synthesis.

Data Extraction Process

The data extraction process for this systematic review was designed to ensure accuracy, consistency, and clinical relevance regarding rituximab's role in treating granulomatosis with polyangiitis (GPA). Initially, two independent reviewers screened articles based on titles and abstracts to determine potential relevance, categorizing each as "relevant," "irrelevant," or "possibly relevant." Articles deemed "relevant" or "possibly relevant" underwent a full-text review, during which data extraction was performed using a custom-designed form in Microsoft Excel (Microsoft Corp., Redmond, WA). Each article was independently assessed by two reviewers according to inclusion and exclusion criteria, with a third senior reviewer consulted in cases of disagreement to ensure data integrity.

The structured data extraction form systematically recorded essential details from each study, including the lead author's name, publication year, study methodology, sample size, key outcomes related to rituximab's efficacy in inducing and maintaining remission in GPA, and potential study biases or limitations. This methodical approach ensured comprehensive and accurate data capture, facilitating a robust synthesis and analysis of evidence to determine rituximab's effectiveness in the clinical management of GPA.

Data Analysis and Synthesis

Due to variability in study designs and outcome measures, we opted against a meta-analysis and focused on a qualitative synthesis to better understand rituximab's effects on remission rates in granulomatosis with polyangiitis (GPA). This approach allowed us to delve into the nuances of each study, considering clinical context and specific outcomes associated with rituximab treatment. We organized the extracted data into thematic categories such as efficacy in induction and maintenance of remission, comparison to other therapeutic agents, and safety profiles. This thematic analysis identified common patterns and discrepancies, providing a clearer understanding of rituximab's role in managing GPA.

Our synthesis was narrative, integrating findings to present a comprehensive overview of the current evidence. We discussed the clinical implications, highlighted gaps in the literature, and proposed future research directions. This qualitative approach not only synthesized disparate findings but also evaluated the strength and reliability of the evidence. By doing so, we aimed to offer insightful conclusions about rituximab's efficacy, enriching the clinical dialogue around its use in GPA and setting the stage for further investigative endeavors.

Results

Study Selection Process

**Figure 1:**
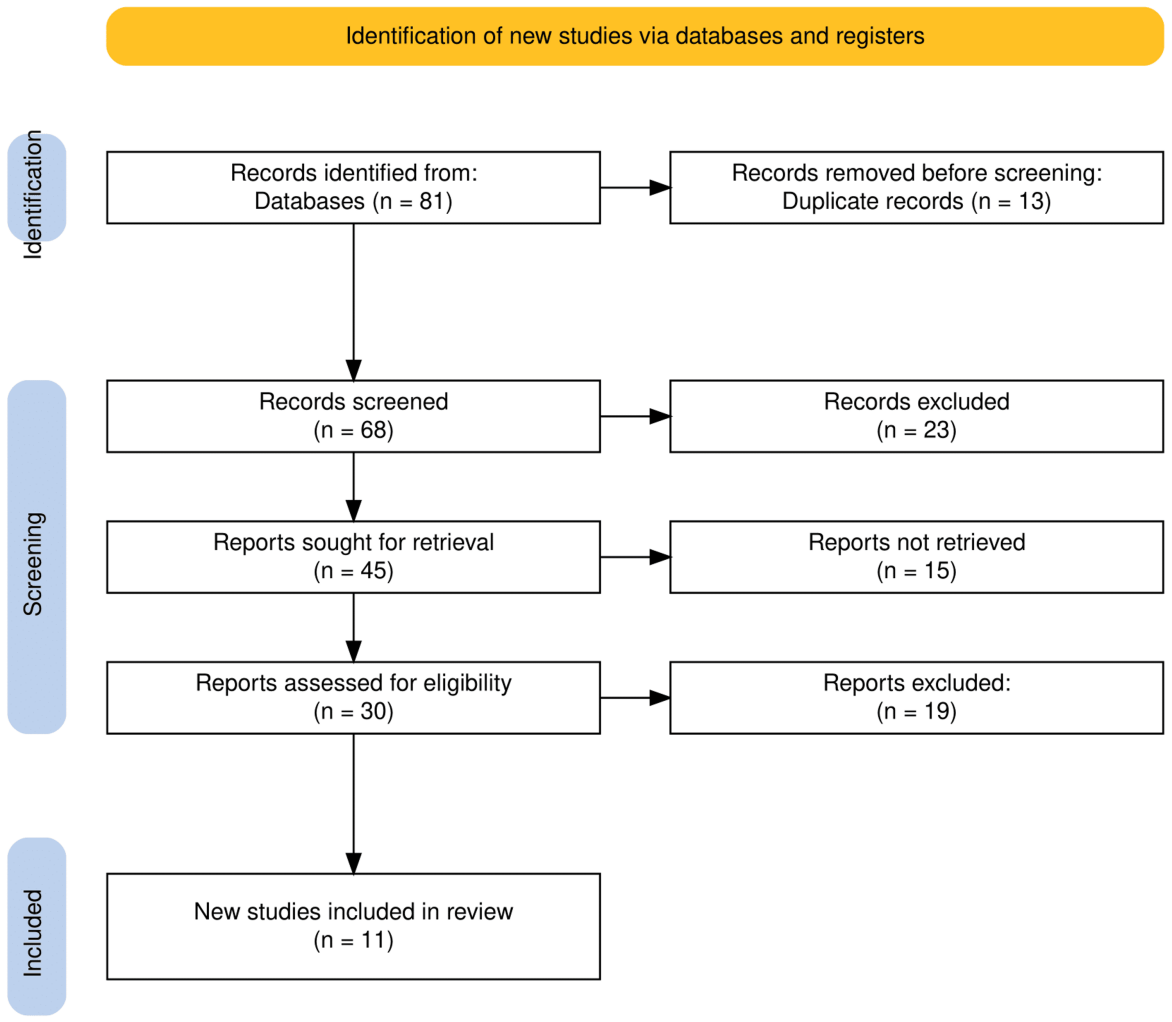
PRISMA flowchart of study selection for systematic review PRISMA: Preferred Reporting Items for Systematic Reviews and Meta-Analyses

The search strategy implemented across several databases initially identified 81 records. After removing 13 duplicate records, 68 unique studies remained for screening. From these, 45 reports were deemed relevant for further detailed evaluation. Subsequently, 30 of these reports were fully assessed for eligibility based on predefined inclusion and exclusion criteria. This thorough review process led to the exclusion of 19 reports, primarily due to non-conformity with the study parameters or insufficient data on the outcomes of interest. Ultimately, 11 studies met all criteria and were included in the final systematic review. The PRISMA flowchart provided in Figure 1 visualizes each step of this selection process, ensuring transparency and replicability of the study methodology.

Characteristics of Selected Studies

The selected studies offer a robust examination of rituximab's efficacy across different contexts of ANCA-associated vasculitis, showcasing diverse methodologies and participant demographics. Starting with Stone et al. [7], a multicenter, randomized, double-blind trial demonstrated that rituximab was not inferior to cyclophosphamide for inducing remission in severe vasculitis and might even be superior in recurrent cases. Following this, Smith et al. [8] in a superiority trial found that rituximab was more effective than azathioprine in preventing relapse in patients with a history of the disease, signifying its potential for long-term management. Furthermore, Charles et al. [9], through a randomized trial, showed that extended biannual rituximab infusions significantly reduced relapse rates compared to standard therapy, underscoring its efficacy in maintaining disease control over time.

Additionally, Jones et al. [10], in their study, evaluated rituximab combined with cyclophosphamide in patients with renal involvement. However, they found no significant difference in composite outcomes of death, renal failure, or relapse compared to the control group. Guillevin et al. [11] further confirmed rituximab's superior performance in maintaining remission at 28 months compared to azathioprine in a similar patient demographic. Contrasting with these larger trials, Seo et al. [12] provided insights into rituximab's effectiveness in a small cohort of patients with limited Wegener's granulomatosis, all of whom achieved remission, emphasizing its potential in refractory cases.

Brogan et al. [13], in a phase IIa trial, demonstrated rituximab's safety and high efficacy in pediatric patients, highlighting its tolerability and the successful management of early-onset or relapsing granulomatosis with polyangiitis and microscopic polyangiitis. Moreover, Wawrzycka-Adamczyk et al. [14] illustrated that lower doses of rituximab could induce remission effectively, presenting a cost-effective treatment option. Keogh et al. [15], in a prospective open-label pilot trial, showed that rituximab was effective and well-tolerated as a remission induction agent in patients with severe refractory Wegener's granulomatosis, with complete clinical remission achieved by three months. Finally, Miloslavsky et al. [16], in a randomized, double-blind, placebo-controlled trial, reported that while rituximab and azathioprine regimens effectively controlled the disease in most patients, approximately one-fourth experienced persistent activity or relapses, suggesting the need for ongoing management strategies to enhance treatment outcomes. Keogh et al. [17] highlighted rituximab's effectiveness in inducing stable remissions in patients with refractory ANCA-associated vasculitis, demonstrating its potential as a mechanism-based treatment. These studies collectively underscore rituximab's transformative impact on the treatment landscape of ANCA-associated vasculitis, providing compelling evidence of its efficacy across different settings and patient subsets. A summary of all the studies is provided in Table 1.

**Table 1:** Summary of selected studies evaluating the efficacy of rituximab in inducing and maintaining remission in patients with GPA ANCA: anti-neutrophil cytoplasmic antibody, GPA: granulomatosis with polyangiitis, MPA: microscopic polyangiitis, AAV: ANCA-associated vasculitis, RTX: rituximab, CYC: cyclophosphamide, AZA: azathioprine, PR3-ANCA: proteinase 3 anti-neutrophil cytoplasmic antibody, IV: intravenous, WG: Wegener's granulomatosis

Lead author and year	Study design	Participants' description	Intervention comparison	Main outcome	Key results	Conclusion
Stone et al. (2010) [7]	Multicenter, randomized, double-blind, double-dummy, non-inferiority trial	197 ANCA-positive patients with either WG or MPA	RTX (375 mg/m²/week for 4 weeks) versus CYC (2 mg/kg/day)	Remission of disease without the use of prednisone at 6 months	64% in the RTX group achieved remission versus 53% in the CYC group; more effective in relapsing disease	RTX was not inferior to CYC for the induction of remission in severe AAV and may be superior in treating relapsing disease.
Smith et al. (2023) [8]	International randomized controlled, open-label, superiority trial	188 patients with relapsing AAV	RTX (1,000 mg every 4 months through month 20) versus AZA (2 mg/kg/day, tapered after month 24)	Time to disease relapse (either major or minor relapse)	RTX superior to azathioprine in preventing relapse 22% versus 36% experienced at least one serious adverse event	Fixed-interval, repeat-dose RTX was superior to azathioprine for preventing disease relapse in patients with a history of AAV relapse.
Charles et al. (2020) [9]	Randomized controlled trial	97 patients with GPA and MPA	RTX or placebo infusion every 6 months for 18 months	Relapse-free survival at month 28	96% and 74% relapse-free survival in RTX and placebo groups, respectively; major relapse-free survival: 100% and 87% for RTX and placebo groups, respectively	Extended therapy with biannual RTX infusions significantly reduced the incidence of AAV relapse compared to standard maintenance therapy.
Jones et al. (2015) [10]	Randomized controlled trial	44 patients with newly diagnosed AAV and renal involvement	RTX (375 mg/m²/week × 4) with two IV CYC pulses versus IV CYC followed by azathioprine	Composite of death, end-stage renal disease, and relapse at 24 months	Composite outcome: 42% in the RTX group versus 36% in the control group; relapses associated with B-cell return	At 24 months, there was no significant difference in the rates of death, end-stage renal disease, or relapse between the two groups.
Guillevin et al. (2014) [11]	Randomized controlled trial	115 patients with newly diagnosed or relapsing GPA, MPA, or renal-limited AAV	RTX (500 mg on days 0 and 14 and at months 6, 12, and 18) versus azathioprine daily until month 22	Rate of major relapse at month 28	Major relapse occurred in 3% of the RTX group versus 29% of the azathioprine group; similar frequencies of severe adverse events	RTX was more effective than azathioprine in maintaining remission at month 28 in patients with ANCA-associated vasculitides.
Seo et al. (2008) [12]	Clinical trial	8 patients with limited WG who were refractory to standard therapies	RTX using a standard lymphoma protocol	Efficacy of RTX in inducing disease remission	All 8 patients achieved remission with RTX; successful retreatment after disease flare or B-cell return	RTX effectively induced and sustained remission in patients with limited WG, including those with refractory disease.
Brogan et al. (2022) [13]	Phase IIa, international, open-label, single-arm clinical trial	25 pediatric patients with new-onset or relapsing GPA or MPA	RTX (375 mg/m² body surface area) plus glucocorticoids once per week for 4 weeks	Assessment of safety, tolerability, pharmacokinetics, and efficacy of RTX	Remission achieved in 56% at 6 months, 92% at 12 months, and 100% at 18 months; mostly mild infusion-related reactions	RTX is well tolerated and effective in pediatric patients with GPA or MPA, with a safety profile comparable to adults.
Wawrzycka-Adamczyk et al. (2014) [14]	Retrospective analysis of clinical trial data	12 patients with refractory (GPA)	Lower than average doses of RTX (median dose = 1 g)	Efficacy of RTX in remission induction	Remission achieved in 11 out of 12 patients (92%); total B-cell depletion observed	Lower doses of RTX effectively induced remission in patients with refractory GPA, offering potential cost benefits.
Keogh et al. (2006) [15]	Prospective open-label pilot trial	10 patients with severe refractory WG (ANCA-positive)	RTX (375 mg/m²) 4 weekly infusions combined with oral prednisone (1 mg/kg/day), tapered over 5 months	Efficacy and safety of RTX for remission induction in refractory WG	All patients achieved complete clinical remission by 3 months; prednisone tapered and discontinued by 6 months	RTX was effective and well-tolerated as a remission induction agent for severe refractory WG.
Miloslavsky et al. (2013) [16]	Randomized, double-blind, placebo-controlled trial	197 patients with AAV	RTX versus CYC followed by AZA	Lack of disease activity without glucocorticoid treatment at 6 months	Remission achieved in 86% within the first 6 months, and 42% failed to achieve primary outcome due to active disease or flares	While treatment regimens effectively control AAV in most patients, active disease persists or recurs in about one-fourth of patients. PR3-ANCA positivity is a risk factor for poor outcomes.
Keogh et al. (2005) [17]	Clinical trial	11 patients with AAV refractory to CYC or with contraindications to its use	RTX infusions and glucocorticoids, with three patients also receiving plasma exchange	Induction of remission by B lymphocyte depletion	All patients achieved remission following treatment; remission maintained while B lymphocytes were absent	RTX effectively induced stable remissions in patients with refractory AAV, highlighting its potential as a mechanism-based treatment.

Discussion

This systematic review synthesizes findings from 11 pivotal studies to assess the impact of rituximab on remission rates in patients with granulomatosis with polyangiitis (GPA). Across diverse clinical settings and patient demographics, the data consistently indicate that rituximab, when compared to traditional therapies such as cyclophosphamide and azathioprine, offers a superior efficacy profile in both inducing and maintaining remission. Notably, studies such as those by Stone et al. [7] and Guillevin et al. [11] highlight rituximab's non-inferiority to cyclophosphamide in induction phases and its superior efficacy in maintenance phases, demonstrating relapse rates significantly lower than those observed with conventional therapies. This is particularly relevant in the context of treatment for relapsing disease, where rituximab not only reduces the frequency of relapses but also potentially ameliorates the severity of relapse episodes.

Furthermore, the review reveals that rituximab's effectiveness transcends age groups, with studies such as that of Brogan et al. [13] indicating robust tolerability and sustained remission in pediatric patients. Such findings are crucial as they expand the therapeutic scope of rituximab beyond adult populations, offering a viable, less toxic alternative for younger patients. However, while the aggregated data robustly support rituximab's clinical efficacy, the variability in dosing regimens across studies, ranging from standard lymphoma protocols to lower, cost-effective doses as in Wawrzycka-Adamczyk et al. [14], suggests a need for standardized treatment guidelines that optimize dosage while minimizing adverse effects.

The effectiveness of rituximab in managing GPA as demonstrated in this review aligns with a growing body of evidence supporting B-cell-depleting therapies in autoimmune diseases [18]. This systematic review corroborates findings from earlier meta-analyses, such as those by Jones et al. [10], which also documented the superior efficacy of rituximab over traditional cyclophosphamide in achieving sustained remission and managing relapses in GPA patients. However, while these past reviews established rituximab's efficacy, our analysis provides further granularity by highlighting its effectiveness across varied dosing regimens and patient demographics, including pediatric populations, an area less explored in previous studies.

Moreover, discrepancies in patient outcomes noted between studies in our review and earlier research can largely be attributed to methodological differences, such as variations in study design, length of follow-up, and the criteria used to define remission and relapse. For instance, earlier studies often utilized the Vasculitis Activity Score for defining remission [19], a method that may not capture patient-reported outcomes as effectively as the newer Patient Reported Outcomes Measures (PROMs) used in more recent trials [20]. Such differences are critical to understand as they influence the interpretation of rituximab's efficacy and underscore the necessity for standardizing outcome measures in future trials to ensure consistency and comparability of results across studies.

The findings from this systematic review suggest significant practical implications for the management of GPA. Rituximab's effectiveness in both inducing and maintaining remission provides a compelling argument for its early integration into treatment protocols, potentially as a first-line therapy in place of or alongside cyclophosphamide [21]. This shift could reduce the exposure of patients to the toxic effects of traditional immunosuppressants, thereby improving long-term patient outcomes and quality of life. For clinicians, the inclusion of rituximab in treatment guidelines would require adjustments in monitoring protocols, given rituximab's different side effect profile, particularly the need for vigilance regarding infusion reactions and immune reconstitution [22]. Ultimately, these findings should encourage healthcare providers to consider personalized treatment approaches based on patient-specific factors such as prior relapse history, age, and overall health, thereby optimizing therapeutic outcomes in patients with GPA [23].

The strength of this systematic review lies in its comprehensive methodology and the rigorous criteria used to select studies for inclusion, which encompass a wide range of clinical trials and cohort studies, thereby providing a robust analysis of rituximab's efficacy and safety. Additionally, the review's scope, covering diverse patient populations and dosing regimens, offers valuable insights applicable to various clinical settings. However, the review is not without limitations. The included studies vary in their methodological quality, with some lacking randomized control or double-blinding, which could introduce bias and affect the generalizability of the findings. Moreover, the heterogeneity in how remission and relapse are defined across studies presents challenges in synthesizing data and may obscure subtler aspects of rituximab's efficacy. Recognizing these limitations is crucial for accurately interpreting the results and guiding future research to address these gaps.

This systematic review unveils crucial insights into rituximab's efficacy in granulomatosis with polyangiitis (GPA), notably demonstrating its potential at varying dosages, including lower doses that could significantly reduce healthcare costs and broaden access to treatment [24]. A particularly novel finding is the drug's efficacy in pediatric populations, challenging existing protocols and suggesting rituximab's broader applicability across different age groups [25]. These insights encourage a re-evaluation of treatment strategies, supporting a shift toward more flexible and economically feasible therapeutic options in autoimmune disease management, extending beyond the traditional adult cohorts typically studied.

Given the findings of this review, future research should focus on randomized controlled trials comparing various rituximab dosing schedules to pinpoint the most effective and cost-efficient regimens [26]. Expanding these trials to include diverse patient demographics, particularly pediatric and elderly patients, would enhance the universality of the results. Additionally, exploring predictive biomarkers for rituximab responsiveness and investigating the molecular mechanisms behind its action in GPA could yield personalized treatment approaches and unveil new therapeutic targets, potentially revolutionizing the management strategies for this challenging autoimmune disorder [27].

## Conclusions

This systematic review conclusively demonstrates that rituximab is an effective and versatile agent for inducing and maintaining remission in patients with granulomatosis with polyangiitis (GPA), presenting a superior alternative to traditional cyclophosphamide-based therapies, especially in relapsing cases. The evidence highlights its efficacy across diverse patient populations, including pediatric groups, and suggests potential benefits of varying dosage regimens that could enhance the accessibility and cost-effectiveness of treatment. Given these findings, clinicians should consider integrating rituximab more prominently into treatment protocols for GPA, and future research should continue to refine its use to optimize outcomes for all patients afflicted with this complex autoimmune disorder.

## References

[1] Panupattanapong S, Stwalley DL, White AJ, Olsen MA, French AR, Hartman ME (2018). Epidemiology and outcomes of granulomatosis with polyangiitis in pediatric and working-age adult populations in the United States: analysis of a large national claims database. Arthritis Rheumatol.

[2] Arzoun H, Srinivasan M, Thangaraj SR, Thomas SS, Yarema A, Lee B, Mohammed L (2022). Recent advancements in the management of anti-neutrophil cytoplasmic antibody-associated vasculitis: a systematic review. Cureus.

[3] Casan JM, Wong J, Northcott MJ, Opat S (2018). Anti-CD20 monoclonal antibodies: reviewing a revolution. Hum Vaccin Immunother.

[4] Mössner E, Brünker P, Moser S (2010). Increasing the efficacy of CD20 antibody therapy through the engineering of a new type II anti-CD20 antibody with enhanced direct and immune effector cell-mediated B-cell cytotoxicity. Blood.

[5] Zhang Z, Xu Q, Huang L (2023). B cell depletion therapies in autoimmune diseases: monoclonal antibodies or chimeric antigen receptor-based therapy?. Front Immunol.

[6] Page MJ, McKenzie JE, Bossuyt PM (2021). The PRISMA 2020 statement: an updated guideline for reporting systematic reviews. BMJ.

[7] Stone JH, Merkel PA, Spiera R (2010). Rituximab versus cyclophosphamide for ANCA-associated vasculitis. N Engl J Med.

[8] Smith RM, Jones RB, Specks U (2023). Rituximab versus azathioprine for maintenance of remission for patients with ANCA-associated vasculitis and relapsing disease: an international randomised controlled trial. Ann Rheum Dis.

[9] Charles P, Perrodeau É, Samson M (2020). Long-term rituximab use to maintain remission of antineutrophil cytoplasmic antibody-associated vasculitis: a randomized trial. Ann Intern Med.

[10] Jones RB, Furuta S, Tervaert JW (2015). Rituximab versus cyclophosphamide in ANCA-associated renal vasculitis: 2-year results of a randomised trial. Ann Rheum Dis.

[11] Guillevin L, Pagnoux C, Karras A (2014). Rituximab versus azathioprine for maintenance in ANCA-associated vasculitis. N Engl J Med.

[12] Seo P, Specks U, Keogh KA (2008). Efficacy of rituximab in limited Wegener's granulomatosis with refractory granulomatous manifestations. J Rheumatol.

[13] Brogan P, Yeung RS, Cleary G (2022). Phase IIa global study evaluating rituximab for the treatment of pediatric patients with granulomatosis with polyangiitis or microscopic polyangiitis. Arthritis Rheumatol.

[14] Wawrzycka-Adamczyk K, Zugaj A, Włudarczyk A, Kosałka J, Sznajd J, Bazan-Socha S, Musiał J (2014). Lower doses of rituximab in remission induction for refractory granulomatosis with polyangiitis. Przegl Lek.

[15] Keogh KA, Ytterberg SR, Fervenza FC, Carlson KA, Schroeder DR, Specks U (2006). Rituximab for refractory Wegener's granulomatosis: report of a prospective, open-label pilot trial. Am J Respir Crit Care Med.

[16] Miloslavsky EM, Specks U, Merkel PA (2013). Clinical outcomes of remission induction therapy for severe antineutrophil cytoplasmic antibody-associated vasculitis. Arthritis Rheum.

[17] Keogh KA, Wylam ME, Stone JH, Specks U (2005). Induction of remission by B lymphocyte depletion in eleven patients with refractory antineutrophil cytoplasmic antibody-associated vasculitis. Arthritis Rheum.

[18] Henderson SR, Copley SJ, Pusey CD, Ind PW, Salama AD (2014). Prolonged B cell depletion with rituximab is effective in treating refractory pulmonary granulomatous inflammation in granulomatosis with polyangiitis (GPA). Medicine (Baltimore).

[19] Kermani TA, Cuthbertson D, Carette S (2016). The Birmingham vasculitis activity score as a measure of disease activity in patients with giant cell arteritis. J Rheumatol.

[20] Kluzek S, Dean B, Wartolowska KA (2022). Patient-reported outcome measures (PROMs) as proof of treatment efficacy. BMJ Evid Based Med.

[21] Shah S, Geetha D (2015). Place in therapy of rituximab in the treatment of granulomatosis with polyangiitis and microscopic polyangiitis. Immunotargets Ther.

[22] Fouda GE, Bavbek S (2020). Rituximab hypersensitivity: from clinical presentation to management. Front Pharmacol.

[23] Springer J, Nutter B, Langford CA, Hoffman GS, Villa-Forte A (2014). Granulomatosis with polyangiitis (Wegener's): impact of maintenance therapy duration. Medicine (Baltimore).

[24] Merkel PA, Niles JL, Mertz LE, Lehane PB, Pordeli P, Erblang F (2021). Long-term safety of rituximab in granulomatosis with polyangiitis and in microscopic polyangiitis. Arthritis Care Res (Hoboken).

[25] Jamois C, Gibiansky L, Chavanne C (2022). Rituximab pediatric drug development: pharmacokinetic and pharmacodynamic modeling to inform regulatory approval for rituximab treatment in patients with granulomatosis with polyangiitis or microscopic polyangiitis. Clin Transl Sci.

[26] Pierpont TM, Limper CB, Richards KL (2018). Past, present, and future of rituximab-the world’s first oncology monoclonal antibody therapy. Front Oncol.

[27] Habibi MA, Alesaeidi S, Zahedi M, Hakimi Rahmani S, Piri SM, Tavakolpour S (2022). The efficacy and safety of rituximab in ANCA-associated vasculitis: a systematic review. Biology (Basel).

